# Rethinking community‐based clinical training in Japan: Toward a more effective model for increasing the number of general practice physicians

**DOI:** 10.1002/jgf2.70038

**Published:** 2025-06-09

**Authors:** Masanobu Okayama

**Affiliations:** ^1^ Division of Community Medicine and Medical Education, Graduate School of Medicine Kobe University Kobe Japan

The effectiveness of Community‐Based Clinical Training (CBCT) in increasing the number of general practice physicians remains uncertain. In Japan, where the population is rapidly aging and shrinking, the demand for general practice physicians has significantly escalated to ensure adequate health care for the public, particularly in rural and underserved areas. General practice physicians are expected to play a vital role in providing comprehensive and continuous care to the aging population. Therefore, finding an effective strategy to secure an adequate number of general practice physicians is a crucial social issue.

CBCT is considered a promising approach to addressing this issue. It provides students and residents opportunities to engage with various aspects of community healthcare, including outpatient care, home visiting care, nurse home care, preventive care, health education, and more, particularly in rural and underserved areas. First introduced in the United States, CBCT has been implemented in numerous countries. It has spread rapidly worldwide since the 1990s, and nearly all medical schools and teaching hospitals in Japan offer this clinical training program.

Several studies have examined the effects of CBCT. Trainees report that their experiences during CBCT profoundly influence their understanding of general practice. They acknowledge the importance of acquiring diverse perspectives, understanding the physician's role, recognizing the significance of community care, respecting individual lifestyles, addressing multimorbidity, navigating various healthcare settings, and collaborating with different professionals.^1^ This training has a positive short‐term impact on attitudes and awareness related to general practice and community healthcare. Participants' ratings of their attitudes and awareness significantly improved after the training, not only in rural areas,^1^ but also in urban settings.^2^

However, the effect of CBCT is insufficient regarding career preferences for general practice physicians. The research conducted with graduates from a single medical university suggests that conventional CBCT might not be sufficient in Japan for effectively developing general practice physicians.^3^ Additionally, a study at one teaching hospital indicated no positive relationship between the amount of compulsory undergraduate education in community‐based medicine and the subsequent rise in the number of residents choosing general practice majors in Japan.^4^

A Longitudinal Integrated Clerkship (LIC) is a novel model of CBCT where students participate in the comprehensive care of patients over time and maintain ongoing learning relationships with both patients and supervising clinicians across multiple disciplines. Although there is no definitive minimum duration for student placements in an LIC program, several months or longer are typically adopted. A LIC provides more extended exposure to community healthcare than conventional CBCT, which lasts 2–4 weeks. This long‐term immersion in a community allows students to become part of the medical and healthcare team and engage deeply with the community. Students in LICs are reported to possess well‐developed patient‐centered communication skills, demonstrate an understanding of the psychosocial contributions to medicine, and express greater preparedness in higher‐order clinical and cognitive skills. They take on increased responsibilities with patients and report feeling more confident in addressing ethical dilemmas. Furthermore, a positive association exists between participation in rural longitudinal integrated clerkship programs and graduates pursuing rural careers and primary care specialties.^5^ The LIC model shows potential; however, further research is needed to determine if these benefits apply to urban settings.

In 2009, the Japan Primary Care Association introduced board certification for family medicine. Since 2018, an independent third‐party organization has managed a new national certification system for the specialty, which includes general practice. Before these actions, there was no board certification for general practice; now, the circumstances surrounding this field have dramatically changed. This situation presents an excellent opportunity to increase the number of general practice physicians. However, the current CBCT may still be insufficient for the effective development of general practice physicians, and several aspects require improvement. Program coordinators must reconsider learning objectives, learning environments, training periods, learning content and activities, learning styles, teaching skills, community interaction, and other relevant factors. Therefore, a more effective CBCT needs to be developed. More research is also required to determine whether CBCT can indeed increase the number of general practice physicians. We hope this ambiguous research question will be resolved in the future.

## AUTHOR CONTRIBUTIONS

**Masanobu Okayama:** Conceptualization; writing – original draft; writing – review and editing.

## CONFLICT OF INTEREST STATEMENT

The author declares no conflict of interest.
